# Generalized eruptive syringomas

**DOI:** 10.1093/skinhd/vzaf096

**Published:** 2025-12-30

**Authors:** Shufang Chai, Aihua Wei Sr, Rong Tao Sr

**Affiliations:** Department of Dermatology, Beijing Tongren Hospital, Capital Medical University, Beijing, P.R. China; Department of Dermatology, Beijing Tongren Hospital, Capital Medical University, Beijing, P.R. China; Department of Dermatology, Beijing Tongren Hospital, Capital Medical University, Beijing, P.R. China

## Abstract

This report describes an unusual clinical case of extensive syringoma involvement spanning multiple anatomical regions, offering insights into diagnostic challenges and management strategies for this rare manifestation.

Dear Editor, A 20-year-old woman presented with a 6-year history of widespread, chronic, asymptomatic and skin-coloured to light-brownish papules. Physical examination revealed symmetrically distributed shiny papules (2–4 mm) (Figure 1a), densely concentrated on the anterior chest and abdomen, with sparse distribution on the extremities (Figure 1b). Histopathological examination revealed dilated eccrine ducts with basophilic epithelial nests in the dermis, featuring a double-layered epithelium and distinctive ­comma-like or tadpole-like ductal configurations (Figure 1c). We diagnosed the patient as having generalized eruptive syringomas. At the 5-month follow-up, lesions remained stable without progression or regression. No treatment was administered.

**Figure 1:**
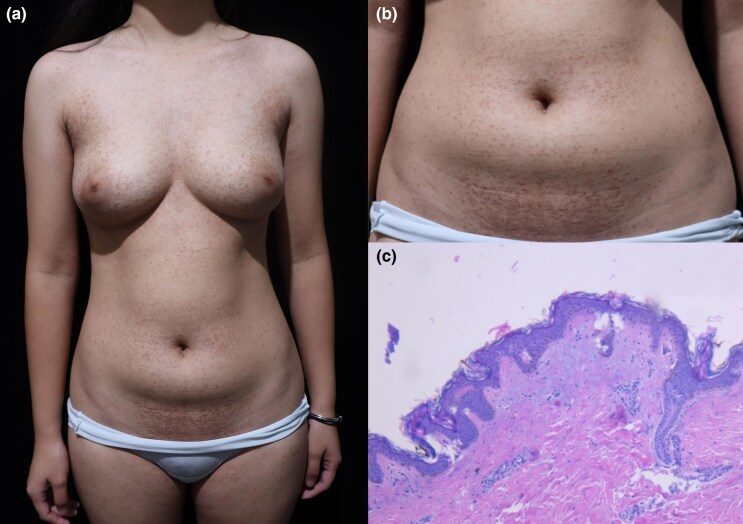
(a) Innumerable skin-coloured to light-brownish papules were symmetrically distributed and most densely concentrated on the anterior chest and abdomen. (b) The lesions had a characteristic shiny appearance, measuring 2–4 mm in diameter. (c) Histopathological examination demonstrated dilated eccrine ducts accompanied by basophilic epithelial cell nests or cords within the dermis (haematoxylin and eosin, original magnification ×40).

## Data Availability

The data underlying this article will be shared on reasonable request to the corresponding author.

